# Effects of the application of sodium ascorbate after in-office bleaching on the penetration of hydrogen peroxide, color change, and microtensile bond strength

**DOI:** 10.1590/0103-6440202305214

**Published:** 2023-12-22

**Authors:** Alexandra Mena-Serrano, María G. Granda-Albuja, Jenny Naranjo, Eliana Aldás Fierro, Michael Willian Favoreto, Alessandro D. Loguercio, Alessandra Reis

**Affiliations:** 1 School of Dentistry, Universidad de Las Américas, Quito, 170125, Ecuador; 2 Laboratorios de Investigación. Universidad de Las Américas, Quito 170125, Ecuador; 3 Department of Restorative Dentistry, School of Dentistry, State University of Ponta Grossa, Brazil

**Keywords:** tooth bleaching, hydrogen peroxide, tooth permeability

## Abstract

This study aimed to evaluate the effects of the application of 10% sodium ascorbate (SA) after in-office bleaching on the penetration of hydrogen peroxide (HP) into the pulp chamber, color change, and microtensile bond strength (µTBS) to the resin-enamel interface. Thirty premolars and thirty molars were randomly divided into three groups (n = 20 each). One group was exposed to deionized water (negative control). The other two groups were bleached with 35% HP in a single session for 3x15 minutes for each application. However, in only one of them, SA was applied for 10 minutes after bleaching. After, the concentration (µg/mL) of HP in each pulp chamber was evaluated by UV-Vis spectrophotometry. Color changes (ΔEab, ΔE00, and ΔWID) were evaluated with a digital spectrophotometer before and in the first week after bleaching. After treatment, molars were restored and sectioned to obtain resin-enamel interface sticks for µTBS at a crosshead speed of 1 mm/min until failure. The HP concentration and µTBS data were analyzed using one-way ANOVA and Tukey tests, and color changes were analyzed by t-tests (α = 0.05). SA application significantly improved the µTBS values and reduced the HP concentrations within the pulp chambers (p < 0.0001). The application of SA significantly interfered with the color changes after bleaching when compared to the control group (p < 0.05). Application of 10% SA after in-office bleaching successfully reduced the penetration of HP into the pulp chamber; however, it decreased color change.

## Resumo

Este estudo teve como objetivo avaliar os efeitos da aplicação do ascorbato de sódio a 10% (AS) depois do clareamento em consultório na penetração do peróxido do hidrogênio (PH) na câmara pulpar, mudança de cor e resistência de união (RU) da interface resina-esmalte. Trinta pré-molares e trinta molares foram divididos aleatoriamente em três grupos (n = 20). Um grupo foi exposto em água deionizada (controle negativo). Os outros dois grupos foram clareados com 35% PH numa única sessão de 3x15 minutos para cada aplicação. Porém, só um grupo recebeu AS durante 10 minutos depois do clareamento. Depois, a concentração (µg/mL) do PH no interior de cada câmara pulpar foi avaliado com espectrofotometria UV-Vis. A mudança de cor (ΔE_ab_, ΔE_00_ and ΔWI_D_) foi avaliada como espectrofotômetro digital antes e depois de uma semana do clareamento. Após de cada tratamento, os molares foram restaurados e seccionados em espécimes com interface resina-esmalte para o teste de RU por microtração a uma velocidade de 1 mm/min até a fratura. Os dados da concentração de PH e RU foram analisados usando ANOVA de uma via e teste de Tukey, e a mudança de cor com o teste t (*α* = 0.05). A aplicação de AS melhorou significativamente a RU e reduziu a concentração de PH na câmara pulpar (*p* < 0.0001). A aplicação de AS interferiu significativamente na mudança de cor depois do clareamento comprado com o grupo controle (*p* < 0.05). A aplicação de SA a 10% depois do clareamento em consultório reduziu significativamente a penetração do PH na câmara pulpar e interferiu na mudança de cor.

## Introduction

In a more globalized world and connected by social networks, people are increasingly concerned with their aesthetics, especially when it involves their smiles. There is, therefore, demand for dental offices to perform tooth bleaching, since the procedure is effective in providing aesthetic benefits for patients. Of the techniques available, in-office bleaching features the advantage of bringing immediate benefits directly after the procedure in comparison to at-home bleaching ^1^. This occurs because this technique uses high concentrations of hydrogen peroxide (HP) (20% - 40%) administered by a professional ^2^.

The whitening effect occurs due to the ability of HP to diffuse through the enamel and dentin ^3^. Through oxidative processes, HP breaks down into reactive oxygen species that interact with organic molecules, breaking their bonds by cleavage ^3^, the most desired effect being the bleaching of the tooth structure.

However, it has been observed that HP can diffuse until the pulp chamber ^4^ ^,^ ^5^, generates oxidative stress in pulp cells ^5^, which causes inflammation ^6^ and reduces cell viability ^7^. This evidence can certainly help to explain undesirable tooth sensitivity after bleaching as reported in several clinical studies ^8^ ^,^ ^9^.

Another adverse effect of the in-office bleaching technique is the reduction in bond strength after bleaching procedures ^10^. The free radicals remaining in the dental substrate after whitening can be responsible for this disadvantage. Delaying the adhesive protocols for 7 or even 14 days is recommended to allow the bond strength values to be restored ^11^.

Several strategies have been tested to diminish or prevent one of these adverse effects. While the use of desensitizers during in-office bleaching is a better strategy for preventing HP penetration ^12^, sodium ascorbate (SA) is the therapy most commonly used to restore the initial bond strength to the tooth surface ^10^. However, needing to use these two substances conflicts with the clinician’s desire for simplification. Interestingly, Lima et al. ^13^ observed that the application of SA reduced the toxic effect of carbamide peroxide on MDPC-23 odontoblastic cells and prevented potential damage to the pulp tissue of teeth in rats subjected to in-office bleaching ^14^. These results seem to indicate that the use of antioxidants could be an interesting option for reducing HP penetration into the pulp chamber.

However, to the best of our knowledge, no study has yet evaluated the penetration of HP into the pulp chamber and the color change of teeth subjected to in-office bleaching by applying SA after the procedure.

Therefore, the objective of the present study was to evaluate the effect of the application of 10% SA after in-office bleaching with 35% HP on the penetration of HP into the pulp chamber, on color change and microtensile bond strength (µTBS) to enamel. The null hypothesis to be tested was that the application of 10% SA after in-office bleaching would not affect the concentration of HP in the pulp chamber, color change, or values of bond strength to enamel.

## Material and methods

This in vitro study was submitted to the local ethics committee and approved according to registration number 00011-UDLA-E-2019. Thirty premolars and thirty molars were donated by patients who underwent tooth extraction at the local university for orthodontic reasons or at the recommendation of a professional.

All teeth were analyzed using a stereomicroscope (Sogeresa BS-185, Spain) with 10x magnification. Teeth with morphological alterations or the presence of enamel cracks were excluded. For premolars, to standardize the buccal thickness of the specimens and to prevent the penetration of HP was influenced, X-ray radiography (Endos ACP, Villa Sistemi Medicali, Buccinasco, Milan, Italy) was performed on each tooth. The central X-ray beam was focused at a 90° angle to the buccal surface of each tooth, and each X-ray was taken with an exposure time of 0.5 seconds and a 30-cm focus-object distance (70 kVp - 7 mA). After exposure, the images were digitally obtained and used to measure buccal tooth thickness (SOREDEX ™ DIGORA ™ Optime, KerrHawe SA, Bioggio, Switzerland). Teeth with enamel-dentin thicknesses less than 2.5 mm or greater than 3 mm were excluded. Next, premolars and molars were randomly assigned to three groups. Teeth in the Control group were treated only with deionized water. Teeth in the other two groups were bleached, after which one group was treated with SA (SA), while the other was not treated (NSA).

### Sample size calculation

The primary result of this study involved quantifying the penetration of HP into the pulp chamber. A previous study ^15^ found that the amount of HP detected in the pulp chamber when teeth were subjected to a 35% HP in-office bleaching procedure averaged 1.156 ± 0.338 µg/mL. Using a two-tailed test with 0.05 alpha and 90% power, to detect a 50% difference between groups, sample sizes of at least eight teeth should be tested in each group. In case of possible losses, two extra teeth were used per group.

### Hydrogen peroxide penetration into the pulp chamber

The roots of each tooth were removed approximately three mm apically to the cementoenamel junction using a low-speed diamond disk under water cooling (Isomet 1000, Buehler Ltd., Lake Bluff, USA). The pulp tissue was removed and rinsed with deionized water. The access to the pulp chamber was expanded carefully with a round bur #1014 (FAVA, SP, Brazil) to allow 25 μL of solution to be introduced using a micropipette (Finnpipette F1, Thermo Fisher Scientific, Madrid, Spain) into the pulp chamber.

This study used analytical products without prior purification, and all solutions were prepared using deionized water. First, a standard reference line was plotted with a 5.000 μg/mL stock solution prepared from a concentrated solution (37% HP, Thermo Fisher Scientific, Madrid, Spain). This solution was diluted in an acetate buffer solution (pH = 4) and calibrated using conventional methods. The solution was titrated with a potassium permanganate solution to determine its analytical grade and the actual solution concentration ^15^. Based on this determined initial concentration, serial volumetric dilutions of 0.000-0.397 μg/mL were performed to plot the standard reference line ^15^. The known concentrations of HP were measured with a UV-Vis spectrophotometer (UV-1280, Shimadzu, CIUDAD, Japan). This procedure yielded a classic reference line for the calculation of the results of the study samples (r = 0.9938).

The specimens were fixed vertically to a wax plate allowing access to the pulp chamber previously treated. The labial area of each specimen was isolated by applying a light-curable resin barrier enclosing an area of 6 mm^2^ (Top Dam, FGM Dental Products, Joinville, SC, Brazil). A 25-μL aliquot of the acetate buffer (pH = 4) was placed into the pulp chamber of each specimen to absorb and preserve all HP that might access the pulp chamber.

To prepare the 10% SA gel, 0.5 mg of SA (98% purity, Sigma-Aldrich Co., St. Louis, MO, USA) was mixed with 5 mL glycerol. The 35% HP bleaching gel was used as an in-office product (Whiteness HP, FGM Dental Products, Joinville, SC, Brazil) in a single session, and it was applied to the enamel buccal surface three times at 15-minute intervals, according to the manufacturer's recommendation. While in one group, 10% SA gel was applied for 10 minutes after the bleaching protocol (SA), in another group, the 10% SA gel was not applied (NSA). For both groups, there was a 10-minute waiting time after bleaching to simulate a time-lapse for the experimental groups before the HP permeability evaluation was carried out. The control group was subjected to deionized water without the use of bleaching agents.

Following the bleaching procedure, the acetate buffer solution in the pulp chamber of each specimen was removed using a micropipette and transferred to a glass tube. To prevent HP from being completely removed, this procedure was repeated by cleaning the pulp chamber of each specimen four times with 25 µL of the acetate buffer and transferring this solution to the same glass tube. Next, 100 µL of 0.5 mg/mL leucocrystal violet (Sigma-Aldrich Chemie GmbH, Steinheim, Germany) along with 50 µL of 1 mg/mL horseradish peroxidase enzyme (Peroxidase Type VI-A, Sigma Chemical Co., St. Louis, MO, USA) and deionized water (2.725 µL) were added sequentially to the glass tube. This sequence was repeated separately for all specimens. The resulting solution was violet with a maximum absorbance peak of 596 nm, which was measured using a UV-Vis spectrophotometer (UV-1280, Shimadzu, Japan). According to Beer’s law, absorbance is directly related to concentration. Therefore, the concentration of HP (μg/mL) was determined by comparing it with the calibration curve already obtained.

### Color change evaluation

A digital spectrophotometer (VITA Easyshade® V, Bad Säckingen, Germany) was used to measure the color change before and one week after the bleaching treatment. During this period, the specimens were immersed in artificial saliva, with temperature controlled at 37 °C. The artificial saliva was changed every day. To measure the initial color of the specimens, guides were constructed with dense condensation silicone (Speedex Putty, Coltene/Whaledent AG, Altstätten, Switzerland) to standardize the position of the spectrophotometer through a 6-mm diameter window with a metal device on the middle third of the buccal surface of each specimen.

The color parameters (L *, a *, and b *) were recorded through the tip of the device inserted in the silicone guide. The L * value represents the lightness (the values varied from 0 for black to 100 for white), the * value represents the color along the red-green axis and the b * value represents the color along the yellow-blue axis. The color change before (baseline) and one week after treatment was given by the difference between the measurements with the spectrophotometer using the CIELab formula ^16^: ∆E_ab_ = [(∆L*)^2^ + (∆a*)^2^ + (∆b*)^2^]^1/2^. In addition, the color change was also calculated based on the CIEDE2000 formula ^17^: ∆E_00_ = [(ΔL/kLSL)^2^ + (ΔC/kCSC)^2^ + (ΔH/kHSH)^2^ + RT (ΔC*ΔH/SC*SH)]^1/2^. The whiteness index (WI_D_) was calculated according to the following formula ^18^: WI_D_=0.551×L−2.324×a−1.1×b. Moreover, changes in WI_D_ caused by each step were calculated by subtracting the values observed at each assessment time from those calculated in the prior step (ΔWI_D_). Perceptual changes were accepted when the differences in the initial and after-bleaching colors present ∆E_ab_ > 2.7 and ∆E_00_ > 1.8 ^19^, and ΔWI_D_ > 2.9 ^20^.

Microtensile bond strength test 

Thirty-sound third molars were used for the µTBS test. The crowns were separated from the roots with a low-speed diamond disc (15HC, IsoMet Diamond Wafering Blades, Buehler Ltd, Lake Bluff, IL, USA) under water cooling, and each tooth on its buccal enamel was ground on wet silicon carbide paper up to 800 grit to create flat surfaces (Norton, Saint-Gobain Peru S.A., Lima, Peru). Then, the teeth were randomly divided into three study groups (Control group, in-office SA, and NSA) according to the in-office bleaching and antioxidant application.

One single calibrated operator performed the adhesive protocol according to the manufacturer’s instructions. The flattened enamel surface received 35% phosphoric acid (Etchant Gel S, Coltène/Whaledent AG, Feldwiesenstrasse Altstätten, Switzerland) for 30 seconds and was rinsed with water for 20 seconds and finally air dried until the appearance of chalky white enamel was evident. Immediately afterward the two-step etch-and-rinse adhesive system (One Coat Bond SL, Coltène/Whaledent AG, Feldwiesenstrasse Altstätten, Switzerland) was applied vigorously for 10 seconds, gently air dried for 5 seconds and light-cured with an LED unit at a constant intensity of 1200 W/cm^2^ for 10 seconds (Bluephase N, Ivoclar Vivadent AG, Bendererstrasse, Schaan, Liechtenstein).

After bonding, the enamel surfaces were restored in three increments of resin composite (Brilliant NG, Coltène/Whaledent AG, Feldwiesenstrasse Altstätten, Switzerland), each 2 mm in height and light-cured for 20 seconds individually with the same LED curing unit. The restored teeth were stored in distilled water at 37 °C for 24 hours before testing. After that, teeth were sectioned with a water-cooled diamond saw (15HC, IsoMet Diamond Wafering Blades, Buehler Ltd, Lake Bluff, IL, USA) at 400 rpm to obtain rectangular sticks of 0.8 mm^2^ area, measured with a digital caliper (CD-15CPX, Mitutoyo, Kanagawa, Japan). The specimens were fixed to a µTBS testing jig with cyanoacrylate resin (Loctite Super Glue Gel Control, Henkel Corporation, Connecticut, United States) and tested in tension at a crosshead speed of 1.0 mm/min using a device operating in µTBS mode (ODEME Biotechnology, Brazil). The failure modes were evaluated under a stereomicroscope at 40x magnification and classified as cohesive, adhesive, or mixed. Premature failures were also registered. Measurements for sticks from the same tooth with adhesive and mixed failure were averaged for statistical purposes.

### Statistical analysis

Before submitting the data for analysis using the appropriate statistical tests, the Shapiro-Wilk test was performed to assess whether the data were normally distributed, and Bartlett´s test for equality of variances was performed to determine if the assumption of equal variances was valid (data not shown). After evaluation of the normal distribution, the data on HP concentration inside the pulp chamber and µTBS were statistically analyzed using one-way ANOVA and the Tukey test, and the data on color change (∆E_ab_, ∆E_00_, and ΔWI_D_) were analyzed by the Student t test (α = 0.05).

## Results

### Hydrogen peroxide penetration into the pulp chamber

One-way ANOVA revealed statistically significant differences among groups (*p* < 0.00001). A significantly lower amount of HP (*p* < 0.00001) was detected in the pulp chamber of the control group than in the bleached groups (Table 1). However, when both bleaching groups were compared, a significantly higher amount of HP (*p* < 0.00001) was observed for the NSA group (Table 1).

**Table 1:** Means and standard deviations of HP concentration (µg/mL) detected in the pulp chamber for all experimental groups (^*‡*^ )

	Control	In-office bleaching	
		NSA	SA
HP concentration	0.00 ± 0.00 A	0.29 ± 0.06 B	0.08 ± 0.02 C

*(* ^*‡*^ *) Different letters indicate statistically significant differences between groups (one-way ANOVA and Tukey test, p < 0.05).*

### Color change evaluation

Student t-tests detected significant bleaching (*p* < 0.02) after one week in both groups (Table 2). However, significant and lower color changes (*p* > 0.07) for all measurements (ΔE_ab,_ ΔE_00_, and ΔWi_D_) were observed for the SA group (Table 2).

**Table 2:** Means and standard deviations of lightness (L*), color along the green-red axis (a*) and color along the yellow-blue axis (b*) at baseline and parameters for color change evaluation for both groups (^‡^)

	Baseline				ΔE_ab_	ΔE_00_	ΔWI_D_
	L*	a*	b*	WI_D_			
NSA	85.9 ± 2.2	0.9 ± 0.5	32.0 ± 3.2	6.6 ± 3.5	8.0 ± 1.6 A	4.9 ± 1.1 a	9.8 ± 2.0 ^a^
SA	85.1 ± 2.1	0.9 ± 0.5	30.7 ± 2.1	7.6 ± 2.0	5.9 ± 0.9 B	3.4 ± 0.8 b	5.7 ± 2.7 ^b^

*(* ^*‡*^ *) Different letters indicate statistically significant differences between groups (Student t-test, p < 0.05). The comparison is only valid in each column.*

### Microtensile bond strength test

Approximately 10-13 resin-enamel bonded sticks were obtained per tooth. The most common fracture mode observed was adhesive/mixed for all experimental groups (Table 3). One-way ANOVA revealed statistically significant differences among groups (*p* < 0.001). No significant difference in µTBS (*p* > 0.05) was observed when the control group was compared with the NSA group (Table 3). However, significantly lower µTBS values (*p* < 0.00001) were obtained in the SA group than in the control and NSA groups (Table 3).

**Table 3:** Fracture pattern, as well as the means and standard deviations of the microtensile bond strength (µTBS, MPa) to enamel for all experimental groups (^*‡*^ )

	Fracture pattern^*‡‡*^			µTBS
	A/M	C	PF	
Control	107 (88.4)	14 (11.6)	0 (0)	28.0 ± 2.8 A
NSA	94 (88.7)	12 (11.3)	0 (0)	21.9 ± 1.6 B
SA	90 (85)	15 (15)	0 (0)	27.6 ± 2.6 A

*(* ^*‡*^ *) Different letters indicate statistically significant differences between groups (one-way ANOVA and Tukey test, p < 0.05). (* ^*‡‡*^ *) A/M = adhesive/mixed failures, C = cohesive failures, PF = premature failures*

## Discussion

Aesthetic treatments involve bleaching and restorative procedures. It is usually recommended to delay restoration for 7 or 14 days after the last whitening session. This time is necessary because the literature ^11^ ^,^ ^22^, as well as the results of the present study, indicate that bond strength values decrease significantly when the restoration is performed immediately after whitening. This adverse effect may be due to free radicals remaining in the dental substrate after the decomposition of HP. These free radicals might interfere with the correct polymerization of the adhesive layer, promoting a drop in its bond strength ^21^ ^,^ ^22^.

Due to its antioxidant properties, SA has been suggested as an alternative to eliminate or reduce residual free radicals from the dental structure postbleaching and improve immediate bond strength values ^10^ ^,^ ^23^ ^,^ ^24^. Our findings confirmed this action, because when 10% SA gel was applied for 10 minutes after in-office bleaching, the bond strength values in enamel were similar to the values in the control group (no bleaching).

However, the most important finding of the present study was an additional advantage of this antioxidant in the prevention of HP diffusion into the pulp chamber. The low molecular weight of the HP molecule allows it to diffuse easily and quickly into the dental substrate, reaching the pulp chamber, which is the main cause of tooth sensitivity. Although the amount of HP inside the pulp chamber depends on certain characteristics of the bleaching products, such as pH ^4^, rheological properties ^25^, composition ^15^, and concentration ^26^, there is a consensus that the presence of a small amount of HP inside the pulp chamber is the most important factor generating tooth sensitivity. Despite these are not consensus regarding the minimum amount of HP to cause deleterious to the dental pulp, as well as which is the maximum amount of HP to be neutralized by the regenerative capacity of the dental pulp. Soares et al. ^5^ showed that when smaller amounts of HP were detected inside the pulp chamber after bleaching, less damage was caused to the pulp cells. Taking this evidence into account, we can think that the use of SA might benefit the pulp tissue viability after in-office bleaching and probably that can be translated to less tooth sensitivity after the treatment. Therefore, future studies should be done to evaluate these hypotheses.

The presence of HP inside the pulp chamber was confirmed in both in-office bleaching groups (SA and NSA), despite a significant difference between them. When SA was applied after bleaching, the HP concentration inside the pulp chamber was significantly lower than that NSA application. According to the authors’ literature search, this is the first study to show that the application of SA significantly decreases the HP content inside the pulp chamber after in-office bleaching. 

SA is an organic sodium salt that is derived from ascorbic acid when a proton from the 3-hydroxy group of ascorbic acid is replaced by a sodium ion ^27^. Its high antioxidant power is due to its ability to act as an electron donor for free radicals, thus limiting oxidative damage ^27^. It is recognized for its high ability to promote the reduction of reactive species derived from oxygen and nitrogen and is thus able to prevent oxidative damage to biological tissue ^28^. Considering that HP is a highly oxidative substance ^3^, the use of SA in association with bleaching procedures has been suggested because SA could protect pulp cells against toxic components in bleaching materials ^13^ ^,^ ^14^.

Lima, Lessa, et al. ^13^, in an in vitro study using a simulated pulp chamber, showed that when SA was associated with bleaching materials, less toxic effects were observed in odontoblast-like cells in comparison with groups in which only bleaching materials were applied. In another study, Lima, Marques et al. ^14^ evaluated whether the oral ingestion of ascorbic acid could prevent the deleterious effects of HP in the pulp cells of rats. The authors showed that pulp regeneration was more rapid when SA was applied. The presence of SA inside the pulp chamber, however, was not measured in either study. The authors of the present study speculate that as SA is a very unstable substance, it may not have penetrated the pulp chamber in these studies. No study was found measuring the amount of SA inside the pulp chamber.

However, as in previous studies ^13^ ^,^ ^14^, the present study showed indirect evidence of the presence of SA inside the pulp chamber. SA has a molecular weight of 198.11 g/mol, close to the molecular weight of potassium nitrate (169.87 g/mol), a compound commonly used to prevent tooth sensitivity induced by bleaching ^29^. It is well known that when applied to enamel, potassium nitrate diffuses through the dentin and reaches the pulp tissue, regardless of concentration or viscosity ^30^.

Therefore, the findings demonstrated in the present study lead the authors to hypothesize that the lower amount of HP measured in the pulp chamber in the SA group could be due to the low molecular weight of this antioxidant. Even though the molecular weight of sodium ascorbate is higher than that of hydrogen peroxide (198.11 g/mol^-1^ and 34.0147^-1^ g/mol, respectively), it is still low enough to allow diffusion through the dental substrate and reduces the final amount of HP after whitening.

These positive results lead us to suggest that from a clinical point of view, SA could cause a reduction in tooth sensitivity induced by bleaching, and future clinical studies need to be performed to prove this hypothesis. However, it is worth mentioning that at least one randomized clinical study evaluated whether the administration of ascorbic acid could prevent bleaching-induced tooth sensitivity ^31^. No significant difference in tooth sensitivity was observed, whether or not SA was used. However, the SA was applied personally, unlike the topical application evaluated in the present study.

Regarding color change, at first glance, the findings of this study show that SA interferes with the bleaching action of 35% HP. As SA diminishes the amount of HP that diffuses through enamel and dentin, there is consequently less HP available to react with the organic structure of the pigments, and thus a lessened whitening effect should be expected, as observed in the present study. However, significant bleaching effects were observed in both groups with ΔE_ab_ (8.0 and 5.9), ΔE_00_ (4.9 and 3.4) and ΔWI_D_ (9.8 and 5.7), mainly because the values of both groups were superior according to Paravina et al. ^19^ to the 50:50% perceptibility and acceptability threshold for ΔE_ab_ (2.7), ΔE_00_ (1.8), and according to Perez et al. ^20^ ΔWI_D_ (2.6).

The mean difference between the groups (2.1 for ΔE_ab_ and 1.5 for ΔE_00_) did not exceed the values of 50:50% perceptibility and acceptability (ΔE_ab_ 2.7 and ΔE_00_ 1.8) (19). This indicates that, despite some significant differences between groups, research findings cannot be fully interpreted in terms of real-life relevance without comparison with perceptibility and acceptability tolerances ^19^. Therefore, considering the perceptibility and acceptability threshold, it is possible to affirm that no clinically relevant differences in the bleaching effect will be expected if SA is associated with in-office bleaching procedures. However, when the mean difference between the groups is evaluated for ΔWI_D_, we find a difference of 4.1 units, this difference exceeds the limits of acceptability and perceptibility > 2.6 ^20^.

As the whiteness index is the most recent and most suitable form of objective evaluation to measure the bleaching level of dental bleaching, which is based on the CIELab color space, this new formula presents a lower probability of error ^18^. WI_D_ showed an improved correlation with the visual perception compared to all others-based whiteness or yellowness indexes tested under laboratory and clinical conditions ^18^ ^,^ ^20^.

It is important to point out that restorative procedures are performed after tooth bleaching, and sometimes 2 to 3 in-office bleaching sessions are necessary. Therefore, SA application may be an interesting option during bleaching sessions to reduce the HP concentration in the pulp and consequently reduce tooth sensitivity. Future studies need to be performed to evaluate this hypothesis.

The application of 10% SA in combination with in-office bleaching with 35% HP can recover the bond strength to enamel and reduce the penetration of HP into the pulp chamber. Although the color change could be affected by SA application, in both groups (AS or NSA application) a clinically important whitening effect was observed.
